# Assessing Flight Angle and Rotor Speed Effects on Drying Efficiency and Power Consumption of the Centrifugal Dryer of Pelletizing Systems

**DOI:** 10.3390/polym17131829

**Published:** 2025-06-30

**Authors:** Mohammadreza Aali, Bernhard Löw-Baselli, Jovan Zecevic, Gerald Berger-Weber

**Affiliations:** Institute of Polymer Processing and Digital Transformation (IPPD), Johannes Kepler University Linz, Altenberger Straße 69, 4040 Linz, Austria; bernhad.loew-baselli@jku.at (B.L.-B.); jovan.zecevic@jku.at (J.Z.); gerald.berger-weber@jku.at (G.B.-W.)

**Keywords:** power and performance efficiency, flight angle, rotor speed, pelletizing systems, drying process, DEM–MPS modeling

## Abstract

This study used the Discrete Element Method (DEM) coupled with the Moving Particle Semi-implicit (MPS) method to investigate the process of drying in the centrifugal unit of a pelletizing system in polymer processing. The effects of various flight angles (10°, 45°, and 70°) and rotor speeds (1280, 1600, and 1920 rpm) on drying efficiency, polymer pellet transport, polymer pellet accumulation, and power consumption were examined. The results showed that the flight angle significantly influenced drying performance. At 1600 rpm, the 10° flight angle configuration required the least power (10.94 kW) but resulted in inefficient water separation, which led to an increase in water droplets (i.e., higher moisture content) in the upper part of the centrifugal unit and near the outlet. With a 70° flight angle, water removal was most effective, but polymer pellet transport efficiency was lower due to centrifugal forces becoming dominant. A 45° flight angle provided the best balance between drying efficiency and power consumption, requiring 16.42 kW while achieving the most efficient polymer pellet transport. Rotor speed also played a crucial role: lower speeds enhanced water removal and reduced power demand but limited throughput, whereas higher speeds facilitated centrifugal separation at the cost of increased power consumption. The optimal combination of the rotor speed and flight angle was found to be 45° at 1280 rpm, which offered an effective trade-off between drying performance and power efficiency.

## 1. Introduction

Pelletization is a crucial preparatory step in plastic production techniques such as injection molding, blown film molding, and extrusion, the last of which is widely used across various industries, including medical, automotive, construction, and food processing industries [1,2,3,4]. Extrusion is a continuous manufacturing process that transforms raw polymeric materials into semi-finished or final products of a desired shape. The downstream parts of the extrusion process, including screw design [5,6], die design [1,3,7,8,9], and the calibration/cooling system [8,10,11,12,13], have been investigated extensively and in detail in numerous studies [8,13,14,15,16,17,18,19,20,21,22,23,24,25,26,27]. However, the upstream processes, particularly the preparation of feed polymers required for various techniques, have attracted less attention in research.

In thermoplastic manufacturing, polymer products are typically produced and distributed as pellets. To enhance its properties, various additives, including stabilizers, pigments, fillers, and reinforcing agents, are incorporated into a polymer melt, which is then shaped into uniform pellets for ease of handling and efficient downstream processing, particularly in extrusion. In polymer manufacturing, three pelletizing methods are mainly used: strand, water ring, and underwater pelletizing. In strand pelletizing, the molten polymer exits the die as a continuous strand, which is then drawn through a cooling bath and cut into cylindrical pellets. In water ring pelletizing, a melt or slurry is extruded through a die and rapidly cooled by a rotating water ring to facilitate solidification and the formation of spherical pellets. Underwater pelletizing, in contrast, involves extruding molten polymer strands into a cutting chamber filled with water, where rotating blades sever the strands into pellets. Within all these systems, water not only cools the freshly cut pellets but also aids in their transport to a centrifugal dryer, a crucial component in which residual moisture is removed by using centrifugal force and airflow to separate water and/or moisture from polymer pellets. Inside the rotating dryer rotor, the pellets experience high-speed motion and interact with flights (angled structures mounted on the rotor walls) that enhance the drying process by improving material transport and particle dispersion. The effectiveness of drying depends on several factors, including the rotational speed of the rotor, the design and angle of the flights, and the interaction forces between the water and pellets. Optimization of this process improves power efficiency and moisture removal, reduces the risk of polymer degradation, and guarantees the overall quality of the final product. Despite the widespread use of centrifugal dryers in pelletizing systems, optimizing their efficiency remains challenging due to their complexity, particularly as they involve multiphase interactions between solid and liquid particles. In fact, most studies in this field have focused mainly on the initial stage of the pelletizing system, where the molten polymers are cut [23,28,29]. Key challenges include (i) inadequate drying, which reduces product quality, and (ii) high power (and consequently energy) consumption, particularly in industrial-scale operations. Pellet agglomeration further reduces drying efficiency and increases processing time. The effects of the design and processing parameters, such as the angle, shape, and location of the flights and rotor speed, on both drying performance and power consumption are not adequately understood, which hinders the development of more efficient drying systems.

Traditionally, optimization of drying processes relied on experimental trial-and-error approaches. These methods required extensive resources, time, and effort and often resulted in inefficient experimental processes and overall designs. The need for physical prototypes, multiple tests, and manual adjustments made this approach not only resource-intensive but also slow and costly.

To overcome the limitations of experimental trial-and-error approaches, computer-aided modeling emerged as a more efficient alternative. Computational Fluid Dynamics (CFD), one of the conventional computer-aided methods, has been used to model the fluid–solid interactions in various engineering applications [30,31,32]. However, its suitability for modeling drying processes, particularly in centrifugal dryers, remains uncertain. CFD methods face challenges in accurately capturing complex particle dynamics, especially in systems with high particle concentrations, where solid–solid and solid–liquid interactions, such as pellet collisions, dispersion, and transport within a rotating environment, play a significant role. As an alternative, the Discrete Element Method (DEM) [14,24], introduced by Cundall and Strack [24], has been increasingly employed to simulate particle behavior in pellets [15,16,17,18,19,20,21,22,33] and multiphase systems [32]. The DEM is a numerical approach that models each individual particle’s motion by solving mechanical and rheological models while taking into account contact forces, friction, and restitution effects. In drying applications, the DEM provides detailed representations of pellet trajectories, residence times, and collision effects, which are crucial for understanding the drying performance inside a centrifugal unit.

In this study, the DEM was employed to model the drying process within the centrifugal unit of a pelletizing system, where both polymer pellets and water droplets are treated as discrete particles. The main objectives of this study were (i) to assess the effectiveness of the DEM coupled with the Moving Particle Semi-implicit (MPS) [14] method (as implemented in the Particleworks software package version 8.1 [34]) in accurately modeling the centrifugal drying process, and (ii) to investigate the effects of critical processing and design parameters, in particular rotor speed and flight angles, on drying efficiency and power consumption. To achieve the first objective, Particleworks, a DEM–MPS-based tool [14], was employed to simulate the complex pellet–water interactions within the centrifugal dryer and evaluate whether the DEM can reliably predict pellet motion and water droplet separation dynamics. The second objective involved simulating various flight configurations and rotor speeds to examine their effects on drying performance and power consumption. Since both flight design and rotational speed play a crucial role in enhancing pellet dispersion and water droplet/moisture separation, optimizing these parameters is essential to improving power efficiency. This study aimed to identify the most power-efficient operating conditions for the centrifugal drying process by systematically comparing power consumption for multiple flight angles at various rotational speeds.

The main contribution of this work lies in the novel application of a coupled DEM–MPS approach to simulate the multiphase dynamics within a centrifugal pellet dryer, with a focus on quantifying power consumption and drying behavior under varying flight angles and rotor speed, an area that has received limited attention in prior studies.

This paper is organized as follows. Section 2 provides a detailed description of the methodology, including the DEM modeling approach, governing equation, simulation setup, variations in flight angles and rotor speeds, and power consumption analysis. Section 3 presents and discusses the results, with a focus on the validation of the DEM for simulating the drying process and analyzing the effects of various flight angles and rotor speeds on process efficiency and power consumption. Section 4 concludes the paper by summarizing the key findings, discussing the implications for pelletizer design, and providing recommendations for future research.

## 2. Methodology

This section outlines the governing equations, approach, and setup used to model the centrifugal drying process of polymer pellets. First, the DEM was employed to simulate the motion and interactions of polymer pellets and water droplets within the drying unit. The section then describes the simulation setup in Particleworks, including the computational model, domain details of the centrifugal unit, boundary conditions, and material properties. Additionally, the methodology covers the analysis of flight angle variations and rotational speed effects on power consumption. This includes the power consumption calculation, the range of flight angles studied, and the evaluation of additional cases with 20% lower and higher rotor speeds for each flight angle. The influence of these processing conditions on drying performance and power consumption is examined.

### 2.1. Governing Equation

The DEM is employed to simulate the motion of solid particles by resolving Newton’s laws of motion for each individual particle. The translational and rotational dynamics of each particle are computed independently based on the following formulations.

**Translational Motion:**

(1) $$m_{i}\frac{{dv}_{i}}{dt}=F_{i}^{C}+F_{i}^{g}+F_{i}^{f},$$

where $m_{i}$ denotes the mass of particle *i*, $v_{i}$ is its velocity, $F_{i}^{C}$ represents the total contact force (including both normal and tangential components) resulting from interactions with other particles or boundaries, $F_{i}^{g}$ is the gravitational force, and $F_{i}^{f}$ accounts for the fluid–particle interaction forces such as drag and buoyancy.

**Rotational Motion:**

(2) $$I_{i}\frac{{d\omega }_{i}}{dt}=T_{i},$$

where $I_{i}$ is the moment of inertia of particle *i*, $\omega_{i}$ is the angular velocity, and $T_{i}$ is the torque generated by tangential forces, including frictional effects at contact points.

The MPS method is utilized to solve the governing equations of incompressible fluid flow, formulated in a Lagrangian framework. The fluid is discretized into particles, and the following Navier–Stokes equations are solved:

**Momentum Equation:**

(3) $$\frac{dv_{i}}{dt}=-\frac{1}{\rho }\nabla p+v\nabla^{2}v+g,$$

where $v_{i}$ is the velocity of fluid particle *i*, ρ  is the fluid density, *p* is the pressure, v is the kinematic viscosity, and *g* represents the gravitational acceleration.

**Incompressibility Constraint:**

(4) ∇·v=0,

In the MPS framework, the spatial differential operators are approximated through weighted summations over neighboring particles, thereby avoiding the need for a computational mesh.

### 2.2. Geometry, Processing Conditions, and Numerical Setup

**Figure 1:**
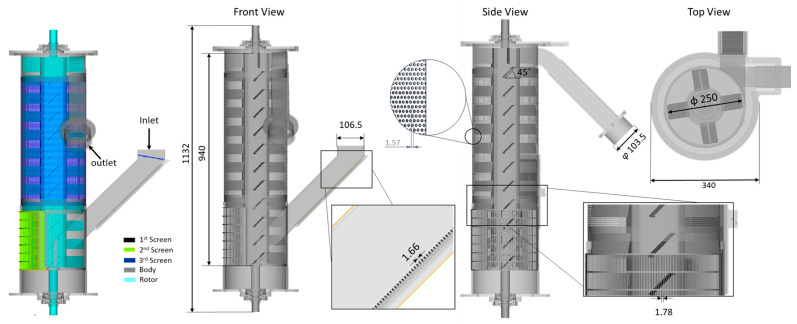
Computational domain and boundary groups (all dimensions are in mm).

In this study, we concentrated on a centrifugal dryer from a pelletizing system as a representative industrial use case in thermoplastic production. Figure 1 illustrates the computational domain, dimensions, and boundary groups, with a flight angle of 45 degrees considered as the reference (REF) case. The rotor operates at a constant rotational speed of 1600 rpm, and centrifugal force, generated by the rotor’s constant angular speed, facilitates particle separation and drying. This ensured analytical consistency and allowed direct comparison of the effects of various flight angles on the drying process and on power consumption. One significant advantage of the Particleworks implementation is that the method is meshless [14,34], which eliminates the meshing step and thus improves efficiency in terms of resource utilization, including preparation time, domain accuracy, and computational resources. The simulation used a Standard Triangle Language (STL) file of the computational domain to ensure accurate geometric representation while simplifying the preprocessing phase. As shown in Figure 1, only the inner part of the centrifugal unit was included in the computational model, while the outer shell (i.e., a protective cover) was ignored. The main function of the screens shown in Figure 1 is to facilitate the removal of excess water from the system, thereby reducing the moisture content of the polymer pellets. This enhances drying efficiency and ensures drier polymer pellets at the outlet.

Water droplets and polymer pellets were modeled as discrete particles with distinct properties. The selection of spherical shapes for both polymer pellets and water droplets was based on their approximate geometrical form observed experimentally, as well as by established conventions in the DEM. This geometric simplification facilitates computational efficiency and model tractability while adequately capturing the essential particle dynamics. The material properties assigned to each particle type are listed in Table 1. The material properties used in this study are based on data from the literature [35,36,37] and values recommended by Particleworks experts. Additionally, certain parameters, such as the spring constant, were obtained through a series of numerical simulations.

Both water droplets and polymer pellets enter the system through the inlet with different throughputs, as specified in Table 2. To ensure a realistic representation of industrial conditions, the material properties and processing parameters were selected based on practical operating values.

The accuracy of the simulation is strongly dependent on the proper regulation of the polymer pellet population within the computational domain. In this study, the number of polymer pellets present in the domain after one second was estimated based on the throughput of both water droplets and polymer pellets. When no particle reduction (0%) was applied, the number of polymer pellets accumulated within the domain exceeded the expected value, leading to an overpopulation of polymer pellets. To address this issue, multiple trials with particle reduction values between 70% and 90% were conducted to determine the appropriate setting that ensures the correct number of polymer pellets remains in the domain over time. While the overall flow patterns remained qualitatively similar, the number of particles introduced into the domain under these conditions did not correspond well with realistic mass flow rates observed in practice. This discrepancy could lead to either an underrepresentation or overrepresentation of particle interactions within the system. In contrast, the 82% reduction provided a particle population that closely matched the expected physical behavior, thereby offering a reasonable balance between computational efficiency and accuracy in capturing the flow dynamics. This setting implies that only 18% of the inlet area is occupied by polymer pellets at each timestep, while no reduction (0%) was applied to water droplets. The assumption underlying this approach is that the inlet is fully filled with water at every timestep, which justifies the absence of particle reduction for the water droplets.

Thermal drying and airflow are indeed significant factors in centrifugal drying and play an important role in determining the overall drying performance. However, in this study, these effects were intentionally omitted, as the main objective was to assess the feasibility and capabilities of the DEM in capturing the drying behavior without the added complexity of fluid and thermal interactions. Accordingly, all simulations were conducted under isothermal conditions. This assumption facilitated a simplified analysis focused on the mechanical separation process while maintaining computational efficiency. However, it should be noted that the isothermal and rigid-body assumptions inherently limit the model’s predictive accuracy. In practical scenarios—particularly those involving soft or partially molten polymer pellets—thermal gradients can lead to spatial variations in viscosity and material deformation, which may influence contact dynamics and overall process behavior. To further enhance computational feasibility, additional simplifications were applied: airflow effects were neglected, as the DEM framework used in this work does not explicitly model fluid flow; water droplet evaporation was not considered due to the isothermal condition; and polymer pellets were modeled as rigid bodies, without deformation or breakage. Further, all particles outside the domain were ignored to streamline the simulation. The simulation time amounted to 5 s. A GPU-based computer was used for the computations, and with the setup presented, running the simulation required approximately two days to complete.

Another important consideration is that, although Particleworks is a meshless method, the quality of the generated computational domain is influenced by the grid interval, which corresponds to the physical spacing used during the import of STL geometry files. A larger grid interval results in coarser surface discretization, which reduces computational cost but may compromise the accuracy of the simulation. Conversely, a smaller grid interval leads to finer surface representation, enhancing accuracy at the expense of increased computation time. The grid interval can be specified either manually or automatically, typically based on the minimum particle size used in the simulation. In this study, a grid interval of 1 mm, corresponding to the diameter of the water droplets, was selected after several trials with both smaller and larger values. This choice represents a compromise between computational efficiency and simulation accuracy.

The timestep (dt) is calculated based on the velocity and size of the polymeric particle and water droplets, given as

(5) $$dt=\frac{Co\times d}{v_{max}},$$

where Co is the Courant number, d is the particle diameter, and $v_{max}$ is the maximum velocity of the particle.

Equation (5) was used to calculate the timestep by taking into account the physical properties of both the polymeric pellets and the water droplets. The final timestep applied in the simulation was chosen as the minimum value obtained from these calculations to ensure numerical stability. Based on this approach, a timestep of 1 × 10^−5^ s was employed in this study.

### 2.3. Analyzing Effects of Variations in Flight Angle and Rotor Speed on Power Consumption and Drying Performance

**Figure 2:**
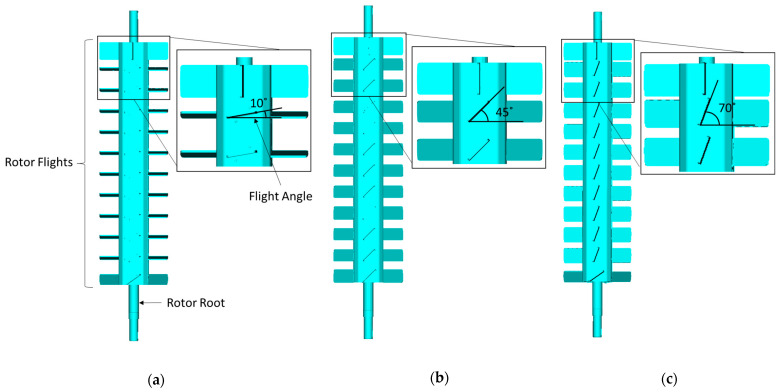
Flight angle configurations analyzed in this study: (**a**) 10°, (**b**) 45° (REF case), and (**c**) 70°.

The centrifugal unit comprised a rotor as illustrated in Figure 1, which was equipped with flight-angled structures that facilitate the transport and distribution of polymer pellets during the drying process. The flight angle plays a crucial role in determining the residence time of the pellets, the frequency of particle collisions, and the overall power efficiency of the drying process. Given the significance of flight angles, their effect on rotor performance was examined by analyzing three configurations, as shown in Figure 2 and indicated in Table 3.

The effects of the processing conditions were examined by simulating additional cases with ±20% variations in rotor speed (relative to the REF case with 1600 rpm) for each flight angle configuration. This analysis aimed to evaluate how changes in rotational speed impact drying efficiency and power consumption. Table 3 presents all simulated cases, including flight angle configurations and corresponding rotor speed variations. Flight angle configurations and rotor speeds were selected for their relevance to industrial drying applications.

This structured approach enabled a comprehensive evaluation of how variations in both flight angle and rotational speed influence the drying process and power consumption. The power required to rotate the rotor was determined based on the torque and angular speed, calculated as

P = T · ω,(6)

where P is the power, T is the torque, and ω is the angular speed.

Equation (6) gives the mechanical power required to overcome pellet interactions and maintain rotor rotation. However, the actual power consumption of the motor is higher due to efficiency losses in bearings, from air resistance, and in electrical components. To determine the electrical power input, we divide mechanical power by motor efficiency. By analyzing the power consumption for all combinations of flight angles and rotor speeds, the most power-efficient configuration was identified, maximizing the drying performance while minimizing power usage.

## 3. Results and Discussion

The Section 3 presents the findings from the simulations and evaluates their implications. First, a qualitative comparison between the numerical modeling results and experimental data is conducted to assess the accuracy and reliability of the DEM–MPS approach in simulating the centrifugal drying process. This is followed by a comprehensive analysis of the effects of different flight angles and rotor speed variations on drying performance, emphasizing their influence on particle behavior, water separation efficiency, the total number of particles, including polymer pellets and water droplets at the outlet, and power consumption. The section concludes with a discussion on power efficiency, identifying the optimal combination of flight angle and rotor speed for achieving the most power-efficient drying process.

### 3.1. Validation of the DEM–MPS Approach to Simulating the Drying Process

**Figure 3:**
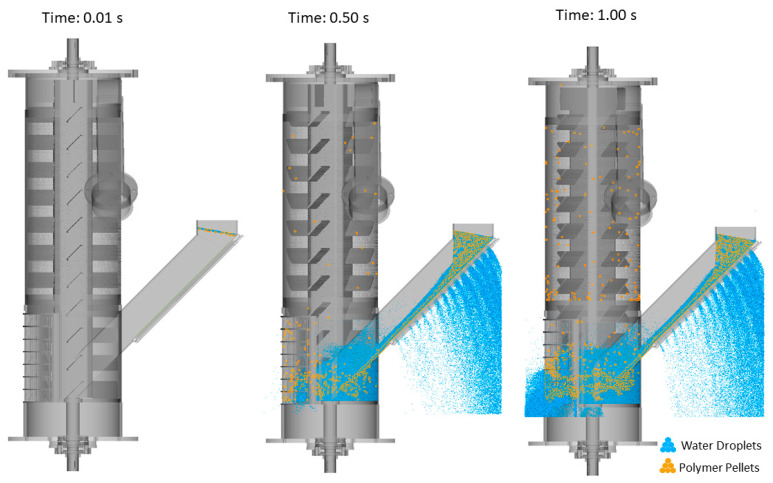
Numerical prediction of the drying process at various stages for the REF case.

The accuracy of the DEM–MPS approach in modeling the drying process inside the centrifugal unit was assessed by analyzing particle behavior and comparing the numerical predictions with experimental data from the industry. Figure 3 illustrates the numerical prediction of the drying process at three sequential stages for the REF case (flight angle of 45° and rotor speed of 1600 rpm), where the particle trajectories demonstrate the separation dynamics, highlighting the effectiveness of the DEM–MPS approach in capturing key drying mechanisms.

In Figure 3, the simulation time was set to 1 s, with the final number of water droplets and polymer pellets reaching approximately 2.1 million and 3000, respectively. Since the main focus of this study was on polymer pellet behavior, the presence of excess water droplets, especially those accumulating outside the domain, was found to be significant. These undesired droplets increased the computational cost and simulation time. To address this issue, the outflow feature in Particleworks was utilized to create three rectangular outflow regions near the first and second screens, where excess water droplets tend to accumulate. This modification effectively reduced the calculation time while preserving the accuracy of the drying process analysis.

**Figure 4:**
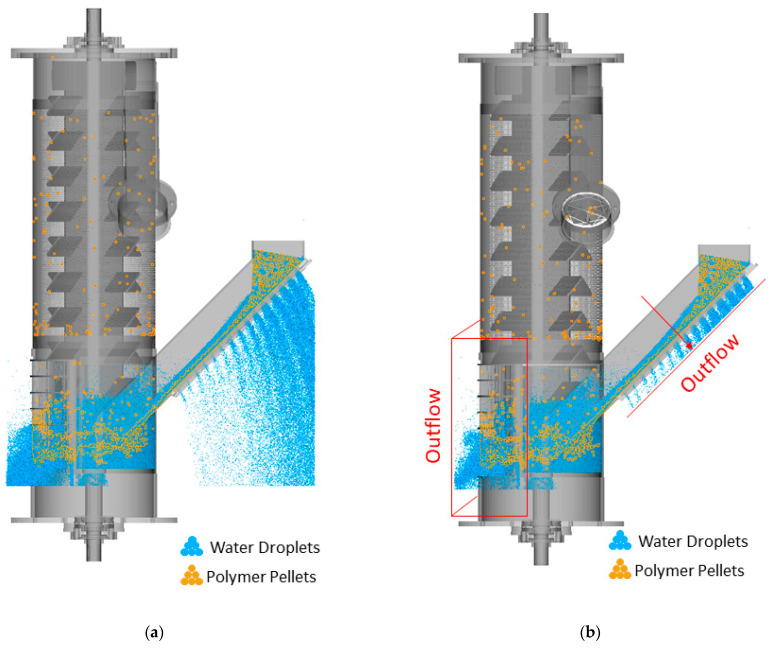
Comparison of the simulation domains for the REF case and a 1 s simulation time: (**a**) original domain without outflow, and (**b**) updated domain with outflow. Red rectangles indicate the outflow regions near the screens.

Figure 4 compares the updated domain, in which outflow regions are near the screens (indicated by red rectangles in Figure 4b), with the original domain without outflows for the REF case and 1 s of simulation time. The results indicate that the inclusion of outflows reduced the number of undesired water droplets considerably from 2.1 million to approximately 1.8 million, consequently decreasing the simulation calculation time from 6 h to 2.5 h. As a result, this approach was adopted in all subsequent simulations.

**Figure 5:**
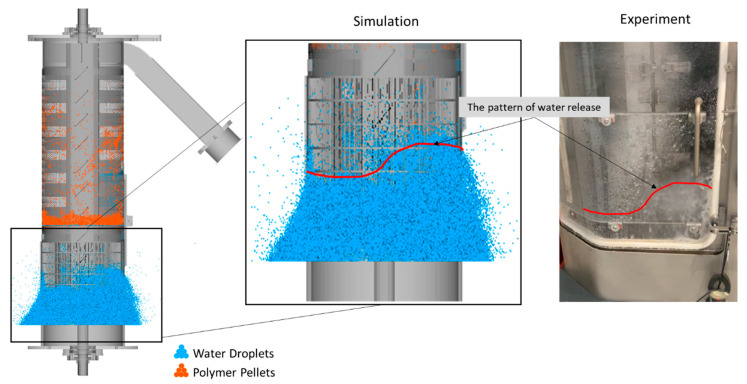
Comparison of simulation predictions with experimental results for the REF case and a 5 s simulation time.

A qualitative comparison with experimental observations, presented in Figure 5, demonstrates that the simulated particle trajectories, distribution, and water droplet release patterns closely correspond to those observed in real drying processes. Due to safety regulations and operational constraints, capturing images of the dryer during operation is not feasible, and thus, obtaining experimental images that correspond directly to the numerical predictions, particularly regarding particle transport behavior along the centrifugal unit, is impossible. However, the available image of an experiment confirms that the simulations effectively capture the efficient separation of water droplets as polymer pellets progress through the centrifugal unit. The simulations successfully replicated key drying mechanisms, including collision dynamics and particle–wall interactions. Notably, in line with experimental observations, most water droplets were released at the second screen, while only a small number of polymer pellets with minimal moisture content reached the third screen. The release of water droplets through the screens in this model is directly influenced by their diameter, with smaller droplets exhibiting a higher likelihood of passing through, thereby increasing water removal efficiency. However, a reduction in particle size also leads to increased computational costs due to a higher number of discrete elements and relative collisions in the simulation. As presented in Table 2, a droplet diameter of 1 mm was selected based on multiple trial simulations to achieve an optimal balance between numerical accuracy and computational efficiency. This ensured a realistic representation of the drying process while keeping the simulation time manageable.

**Figure 6:**
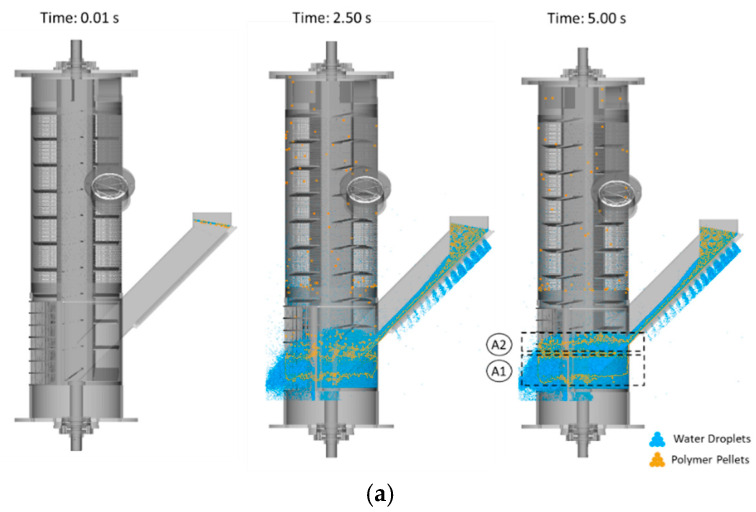

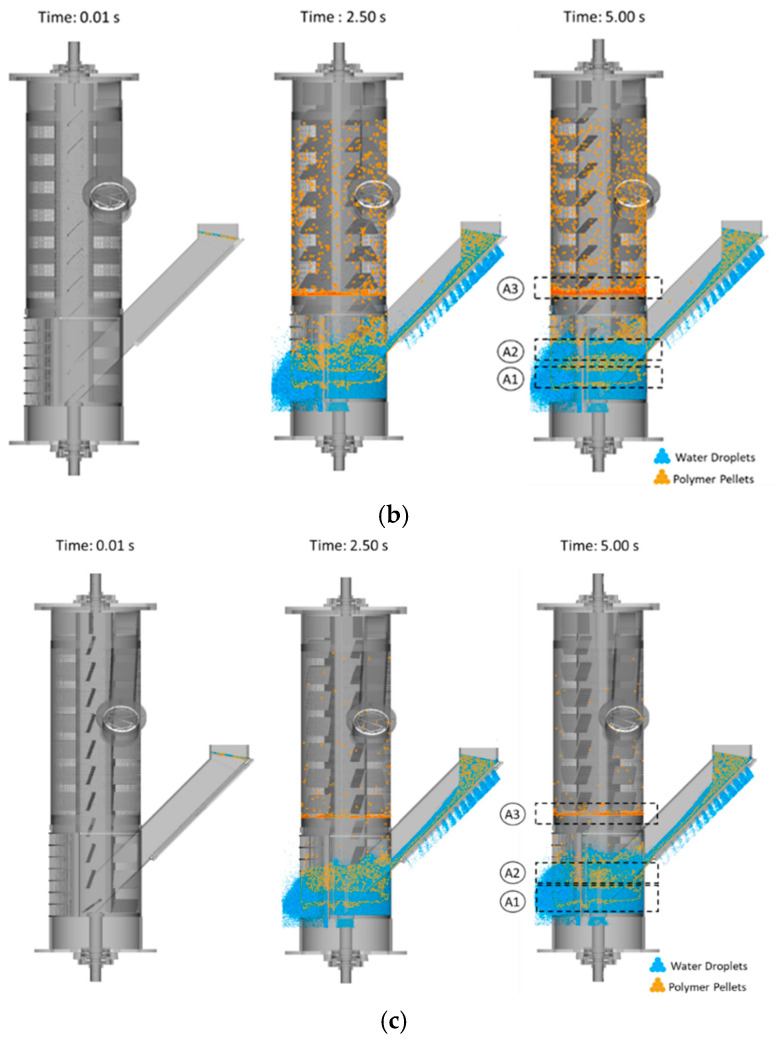
Several stages of the drying process for various flight angles: (**a**) 10°, (**b**) 45° (REF), and (**c**) 70°.

Additionally, the clustering of polymer pellets observed at the top section of the third screen, as shown in Figure 6b, is consistent with experimental findings, particularly for smaller-diameter polymer pellets.

Moving and transforming polymer pellets as they were leaving the centrifugal dryer involved several key stages. Initially, a particular number of polymer pellets, accompanied by a large volume of water droplets, entered the centrifugal unit. Due to the high rotational speed of the rotor, the particles were propelled towards the screen walls, where they continued to rotate. As a result of the particle–particle and particle–wall interactions modeled by the DEM–MPS approach, the polymer pellets gradually detached from the surrounding water droplets and were transported upward through the centrifugal unit before being discharged from the dryer. Additionally, accumulation of polymer pellets was observed in two distinct zones: (i) at the bottom of the centrifugal dryer, between the first and second and between the second (indicated as A1 in Figure 6) and third rows of flights (indicated as A2 in Figure 6), and (ii) at immediately below the third screen (indicated as A3 in Figure 6), where the lower part of the centrifugal body connects to the screen. The first accumulation of polymer pellets occurred due to the strong adhesion between polymer pellets and water droplets in regions with a high water volume. The second accumulation is attributed to a change in domain geometry, as the third screen extends into the lower part of the cylinder. This structural discontinuity led to the accumulation of polymer pellets in the transition zone.

### 3.2. Effect of Flight Angle on Process Efficiency

The effect of the flight angle on the drying process was investigated by analyzing three different configurations, as indicated in Table 3. Figure 6 presents the drying process at various sequential stages for each case study.

The overall functionality of the centrifugal dryer remained consistent across all case studies, with particle movement and water separation following similar trends. However, considerable differences in drying efficiency were observed. Water droplet separation was found to be most efficient in the 70° case, followed by the 45° case and the 10° configuration. As seen in Figure 6a, water droplets reached up to the middle of the centrifugal unit in the 10° case, which indicates inefficient drainage. This resulted in polymer pellets with higher moisture content at the outlet, which is undesirable for drying efficiency.

As illustrated in Figure 6, the first accumulation of polymer pellets was most pronounced in the 10° case (Figure 6a), followed by the 45° (Figure 6b) and 70° (Figure 6c) configurations. High water retention at lower flight angles increased the cohesive forces between polymer pellets and water and thus led to greater material accumulation. As the number of adhering polymer pellets reached a critical threshold, they detached and continued to travel along the centrifugal height. The distances between the rows of flights were larger for 10° than for 45° and 70°. In contrast, the second accumulation of polymer pellets was most pronounced with 45° flights, less with 70°, and almost negligible with 10° flights. The relatively higher material transport efficiency in the 10° case reduced the chances of pellet accumulation at this location. These findings are consistent with experimental observations and thus validate further the accuracy of the simulation.

**Figure 7:**
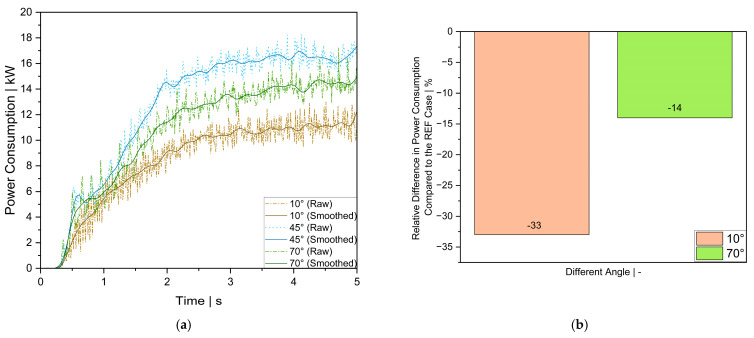
Simulated power consumptions for various flight angles; (**a**) raw and smoothed data, and (**b**) relative variations in power consumption for each case compared to the REF case.

To assess the power requirements for each case studied, the total torque acting on the rotor was computed using Particleworks. The mechanical power consumption was then determined using Equation (6). As observed in Figure 7, the power consumption curve exhibits significant oscillations, making direct comparisons between the individual cases challenging. To clarify the differences in power consumption, the torque data obtained from the simulations were processed using a second-order Butterworth filter [38]. This method effectively reduces high-frequency fluctuations while preserving the essential characteristics of the original signal, thereby ensuring that key trends and variations remain accurately represented. The filter parameters were selected based on preliminary spectral analysis and are as follows: cutoff frequency = 6 Hz, filter order = 2, and sampling frequency = 100 Hz. Figure 7a presents the resulting smoothed power consumption curves.

For improved comparison, the relative variations in power consumption for each case within the stabilization period (3–5 s simulation time) and the REF case are shown in Figure 7b. The stabilization period of 3–5 s was selected based on the observed power consumption trends across all cases, during which the system reached a quasi-steady state characterized by minimal transient effects. This interval was chosen to ensure that comparisons are made under stabilized operating conditions, thereby excluding the influence of initial startup transients. The mean power consumption values were calculated over this stable time interval and then compared to the REF case.

As illustrated in Figure 7a, the power consumption profiles exhibit similar trends across all three case studies. The lowest mean power consumption of 10.94 kW within the stable zone was observed for the 10° flight angle configuration, followed by the 70° configuration with 14.09 kW, and the REF case with the highest power demand of 16.42 kW. Figure 7b also highlights the variations in power consumption relative to the REF case, showing reductions of 33% and 14% for the 10° and 70° cases, respectively.

We attribute the lower power consumption in the 10° case to the smoother particle motion and reduced resistance forces. Conversely, the REF and 70° configurations exhibited increased power demand due to higher water resistance and material accumulation. However, as illustrated in Figure 6, polymer pellet transport was more efficient in the REF case than in the 70° case. This finding deviates from our initial expectations, as improved water separation in the 70° configuration should theoretically enhance polymer transport. The results indicate that, while water separation was more effective in the 70° case, the polymer pellets’ residence time was longer than in the REF case. This suggests that the improved drainage efficiency in the 70° configuration led to greater interactions between the polymer pellets and the unit walls, increasing frictional resistance and extending their transport trajectory within the centrifugal dryer.

Compared to the REF and 70° cases, in the 10° configuration, the amplitude and frequency of the power consumption curve were significantly higher, particularly before the stable zone (up to a 3 s simulation time). This was due to higher instability in the early phase caused by delayed water separation and increased turbulence. The higher amplitude variations suggest irregular particle interactions with the rotor and screens, which may contribute to mechanical wear and potential maintenance issues. Conversely, in the REF and 70° cases, the amplitude and frequency variations were less pronounced, indicating more stable flow interactions and reduced mechanical stress on internal components.

### 3.3. Effect of Rotor Speed on Process Efficiency

As mentioned earlier, rotor speed is one of the key processing parameters and affects both drying efficiency and power consumption. Increasing the rotor speed generally increases centrifugal forces, promotes water separation, and accelerates polymer pellet movement towards the outlet. However, excessive rotor speeds can also lead to higher power consumption due to increased resistive forces and particle collisions.

**Figure 8:**
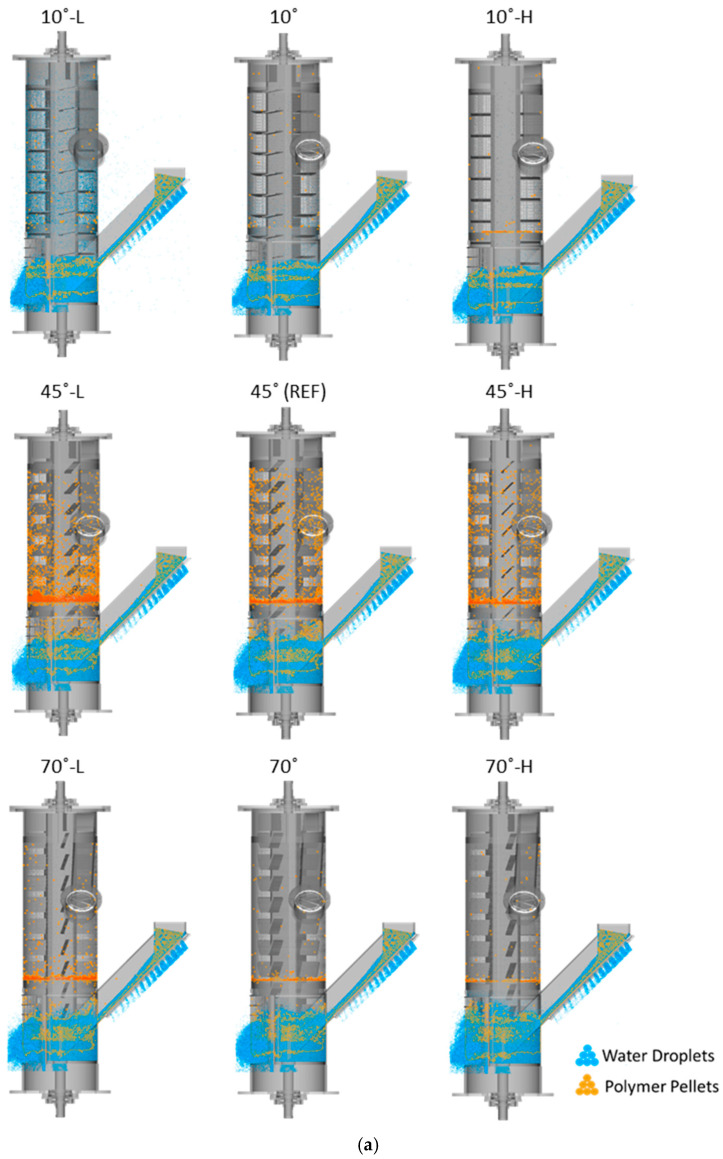

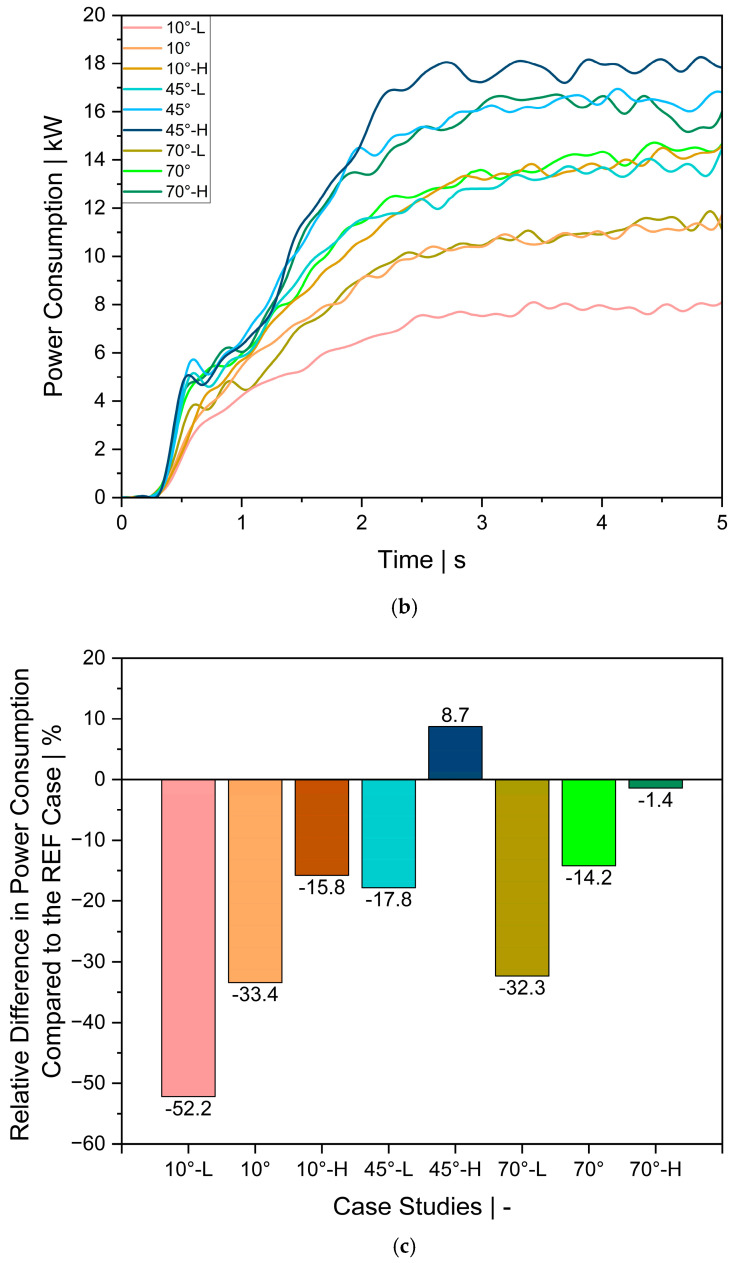
Comprehensive analysis of drying performance and power consumption across all cases studied: (**a**) schematic representation of the drying performance at a 5 s simulation time, (**b**) power consumption, and (**c**) relative differences in power consumption compared to the REF case.

To systematically evaluate the effect of the rotor speed, additional simulations were performed at ±20% of the reference speed for all flight angles (10°, 45°, and 70°), as indicated in Table 3. Figure 8 shows schematics of the drying process at a 5 s simulation time (Figure 8a), the power consumption profiles for various cases (Figure 8b), and the relative variations in power consumption compared to the REF case (Figure 8c).

As can be seen in Figure 8a, the transferring of water droplets toward the outlets was most pronounced in the cases with a 10° flight angle, with the highest degree of transformation observed in the 10°-L case, which exhibited low polymer pellet transformation and consequently no accumulation of polymer pellets. In contrast, in the cases with a 45° flight angle, the transformation of polymer pellets was more efficient, resulting in a near absence of water droplets above the second screen. However, a reduction in the second accumulation of polymer pellets was observed with increasing rotor speed in these cases. In the cases with a 70° flight angle, it was evident that increasing the rotor speed did not improve water separation, as the level of water accumulated in the centrifugal unit increased with higher rotor speeds. However, while the accumulation of polymer pellets remained nearly identical between the 70° and 70°-H cases, water droplet accumulation was notably more pronounced in the 70°-L case, a phenomenon not observed in the 70° and 70°-H cases. Additionally, polymer pellet transformation in these cases remained inefficient, as outlined in Section 3.2. As in the 45° cases, no water droplets were observed above the second screen in the 70° cases.

As shown in Figure 8b, the power consumption profiles for various rotor speeds and flight angles follow similar trends across all cases. The same data smoothing method as mentioned above was applied to all cases. The results presented in Figure 8c demonstrate that changes in rotor speed (both an increase and decrease) had a significant effect on power consumption, but the extent of variation did not follow a consistent pattern relative to changes in rotor speed. The 45°-L case resulted in a 17.80% reduction in power consumption, whereas the 45°-H yielded only an 8.70% increase. Similar trends were observed for the 10° and 70° cases: the 10°-L configuration resulted in a 52.20% reduction, while the 10°-H yielded a 15.80% decrease, and the 70°-L configuration showed a 32.30% reduction, whereas the 70°-H yielded only a 1.40% decrease.

### 3.4. Relevance of the Parameters Studied

This section evaluates the influence of the parameters studied on power consumption and process efficiency. To this end, the total number of polymer pellets and water droplets at the outlet, as presented in Table 4, along with the power consumption data shown in Figure 8, were analyzed.

The results presented in Table 4 demonstrate that the total number of polymer pellets and water droplets at the outlet varied significantly with the flight angle. For the cases with a 10° flight angle, the total number of polymer pellets that reached the outlet was considerably low. Specifically, in the 10° case, only one polymer pellet exited the system, whereas in the 10°-L case, no polymer pellets reached the outlet, although a significant number of water droplets were present. This suggests inefficient drying, as a substantial amount of moisture was retained by the pellets. While power consumption was significantly lower in these cases—with a 52.2% reduction in the 10°-L case and a 15.8% reduction in the 10°-H case—the high moisture retention negatively impacted overall process efficiency, making these configurations suboptimal.

For cases with a 45° flight angle, the results indicate a more favorable balance between drying performance and power efficiency. The 45°-L case exhibited a 17.8% reduction in power consumption compared to the REF case, while the 45°-H case required 8.7% more power. Importantly, the total number of polymer pellets at the outlet was the same for both the 45°-L and REF cases (19 polymer pellets), which indicates that it was possible to achieve effective drying without compromising polymer pellet discharge. These findings suggest that the 45°-L configuration is the most efficient, as it optimally balances power (energy) savings and drying effectiveness.

In contrast, the cases with a 70° flight angle exhibited no polymer pellet outflow, which indicates that, while water separation was efficient, excessive moisture removal adversely affected material discharge. The reductions in power consumption were also less pronounced, with the 70°-L and the 70°-H cases showing decreases of 32.3% and only 1.4%, respectively. The absence of polymer pellet transport at the outlet further suggests that this configuration is not ideal for maintaining process efficiency.

The findings presented provide robust evidence that the cases with a 45° flight angle, particularly the 45°-L configuration, are the most efficient. This is indicated by the effective transport of polymer pellets and the absence of excessive water droplets at the outlet. These results highlight that optimizing both flight angle and rotor speed is key to achieving an optimal balance between drying performance and power efficiency.

## 4. Conclusions

This study employed the Discrete Element Method (DEM) coupled with the Moving Particle Semi-implicit (MPS) method to investigate the drying process within the centrifugal unit of a pelletizing system. Our analysis focused on the effect of the flight angle and rotor speed on drying efficiency, polymer pellet transport, and power consumption.

The results obtained substantiate the practical applicability of the DEM–MPS coupling method implemented in Particleworks for simulating the intricate interactions between polymer pellets and water droplets within a centrifugal dryer. The simulated particle dynamics, including transport under centrifugal forces, droplet detachment, and material accumulation, exhibit strong qualitative agreement with the expected physical behavior of such systems. While direct experimental validation was beyond the scope of the present study, the consistency of the simulated outcomes with known physical phenomena supports the credibility of the proposed modeling approach.

The results demonstrate that flight angles significantly influenced drying efficiency and power consumption. At a constant rotor speed of 1600 rpm, the 10° flight angle configuration consumed the least power (10.94 kW) due to smoother particle motion and reduced resistance forces. However, inefficient water separation was observed, causing water droplets to travel further up to the third screen. Conversely, the 70° flight angle configuration showed the highest water separation efficiency, but polymer pellet transport efficiency was significantly reduced, as indicated by the absence of polymer pellets at the outlet. We attribute this to the predominant influence of centrifugal forces, which directed the particles towards the lateral walls rather than facilitating their discharge. Consequently, this configuration led to prolonged residence time and higher power dissipation (14.09 kW). In contrast, the 45° flight angle configuration exhibited the most favorable balance between drying efficiency and power consumption, enabling a total outflow of 19 polymer pellets. However, this configuration required the highest power input (16.42 kW) due to increased material accumulation and water resistance.

Polymer pellet accumulation was also significantly affected by flight angles. The first accumulation, attributed to strong adhesion between polymer pellets and water droplets, was most pronounced in the 10° flight angle case due to excessive gaps between flights. Increasing the flight angle to 45° and 70° reduced this accumulation and improved drying performance. The second accumulation, which occurred near the third screen, was negligible in the 10° and 70° configurations but more prominent in the 45° cases.

Further, the effect of the rotor speed was evaluated. Lower rotor speeds extended the residence time of polymer pellets, enhancing water removal but increasing the risk of material accumulation. Higher speeds improved centrifugal separation but led to pellet carryover and reduced drying efficiency. At lower speeds, power consumption was minimized, as observed in the 10° (7.85 kW at 1280 rpm), 45° (13.50 kW at 1280 rpm), and 70° (11.12 kW at 1280 rpm) flight angle cases. In contrast, at higher speeds, power demand significantly increased, reaching 13.83 kW for the 10° case, 17.85 kW for the 45° case, and 16.19 kW for the 70° case at 1920 rpm. Thus, an optimal combination of rotor speed and flight angle is necessary to balance efficient water removal, high throughput, and power consumption.

Based on the findings, the 45° flight angle case at 1280 rpm demonstrated the most efficient performance, balancing water separation, polymer pellet transport (19 pellets at the outlet without water droplets), and power consumption. Although its power demand was higher than in the 10° and 70° configurations, it provided the best overall drying efficiency with moderate power requirements.

Additionally, power consumption fluctuations were notably higher in the 10° configuration during the transient phase, indicating an unstable flow regime with irregular particle impacts and turbulence. In contrast, the 45° and 70° cases exhibited more stable power patterns, which can prompt the reduction in mechanical stress and wear on the components. This suggests that higher flight angles contribute to the enhanced stability and durability of the centrifugal unit.

This study underscores the critical role of selecting an optimal combination of the flight angle and rotor speed to enhance drying efficiency while minimizing power consumption in centrifugal pellet dryers. Ongoing research efforts by the authors focus on extending the simulation duration to achieve a steady-state condition, where particles are continuously introduced at the inlet and discharged at the outlet, ensuring a more comprehensive analysis of the drying process. Furthermore, current investigations are directed toward optimizing performance and energy consumption by refining rotor design and exploring advanced flight angle configurations. These efforts aim to develop more efficient and sustainable drying solutions for polymer processing applications.

## Figures and Tables

**Table 1:** Material properties of water droplets and polymer pellets.

Property	Polymer Pellets	Water Droplets
Particle Shape	Sphere	Sphere
Density (kg/m^3^)	1380	1000
Specific Heat (J/kg·K)	450	4200
Young’s Modulus (Pa)	2 × 10^9^	-
Poisson Ratio	0.4	-
Yield Stress (GPa)	0.055	-
Spring Contact	800	-
Contact Angle (°)	90	60
Kinematic Viscosity (m^2^/s)	-	3.639 × 10^−7^
Coefficient of Surface Tension (N/m)	-	0.072
Coefficient of Friction	0.4	0.3
Coefficient of Restitution	0.6	0.2

**Table 2:** Operating conditions used in the numerical modeling.

Property	Polymer Pellets	Water Droplets
Diameter (mm)	3	1
Particle Speed at Inlet (m/s)	0.67	-
Water Throughput at Inlet (m^3^/s)	-	0.0025
Particle Reduction at Inlet (%)	82	0

**Table 3:** Simulated cases, including flight angle variations and rotor speed modifications.

Case	Flight Angle (°)	Rotor Speed (rpm)
10°-L	10	1280 (−20% REF)
10°	10	1600
10°-H	10	1920 (+20% REF)
45°-L	45	1280 (−20% REF)
45° (REF)	45	1600
45°-H	45	1920 (+20% REF)
70°-L	70	1280 (−20% REF)
70°	70	1600
70°-H	70	1920 (+20% REF)

**Table 4:** Total number of polymer pellets and water droplets at outlet after 5 s of simulation time.

Location	Outlet	
Case	Polymer Pellets	Water Droplets
10°-L	0	111
10°	1	0
10°-H	1	0
45°-L	19	0
45° (REF)	19	0
45°-H	10	0
70°-L	0	0
70°	0	0
70°-H	0	0

## Data Availability

The original contributions presented in this study are included in the article. Further inquiries can be directed to the corresponding author.
